# Communication and medical errors: a bibliometric analysis of research trends and knowledge structures

**DOI:** 10.3389/fmed.2026.1856744

**Published:** 2026-08-28

**Authors:** Birgit Hladschik-Kermer, Olena Litvinova, Gernot Gerger, Michał Ławiński, Maima Matin, Maria Kletecka-Pulker, Eva Hollmann, Alina Volkmar, Magdalena Eitenberger, Atanas G. Atanasov, Thomas Wochele-Thoma

**Affiliations:** 1 Unit Medical Psychology, Department of Public Mental Health, Center for Public Health, Medical University of Vienna, Vienna, Austria; 2 Ludwig Boltzmann Institute Digital Health and Patient Safety, Medical University of Vienna, Vienna, Austria; 3 Department of Management, Marketing and Quality Assurance in Pharmacy, National University of Pharmacy of the Ministry of Health of Ukraine, Kharkiv, Ukraine; 4 Department of Biotechnology and Nutrigenomics, Institute of Genetics and Animal Biotechnology of the Polish Academy of Sciences, Magdalenka, Poland; 5 Department of General, Gastroenterology, and Oncologic Surgery, Medical University of Warsaw, Warsaw, Poland; 6 Institute for Ethics and Law in Medicine, University of Vienna, Vienna, Austria; 7 Faculty of Social Sciences, Institute for Political Science, University of Vienna, Vienna, Austria; 8 Department of Biochemistry, Saveetha Medical College and Hospital, Saveetha Institute of Medical and Technical Sciences, Chennai, Tamil Nadu, India

**Keywords:** artificial intelligence, communication, digitalization, healthcare, medical errors, patient safety, teamwork

## Abstract

**Background:**

Communication is increasingly positioned in patient safety research as a cross-cutting domain influencing multiple care processes. However, its structural organization within the scientific literature remains insufficiently integrated.

**Objective:**

This study aims to conduct a large-scale bibliometric analysis of the scientific field linking communication and medical errors and to propose a conceptual communication governance framework integrating the identified communication domains into a coherent structure relevant to healthcare, including nursing practice.

**Methods:**

We conducted a bibliometric analysis of 13,431 publications indexed in the Web of Science Core Collection (search date: 21 February 2026). Publication trends, thematic evolution, keyword co-occurrence, and international collaboration networks were analyzed using Web of Science analytical tools, the bibliometrix R package, and VOSviewer. To ensure robustness, results were cross-validated with an equivalent Scopus dataset.

**Results:**

Publication activity increased exponentially after 2000, exceeding 1,000 publications annually after 2020. Four developmental phases were identified: clinical-descriptive (1964–1999), systemic patient safety (2000–2010), team- and patient-centered (2011–2019), and digital and hybrid communication (2020–2026). Five major thematic clusters emerged: pharmacotherapy safety, teamwork and human factors, information transfer and patient-centered communication, digital infrastructure, and organizational safety culture. The United States, England, and Australia dominated scientific output, with contributions from 139 countries/regions overall. Communication is embedded in multiple patient safety areas across the knowledge network. Cross-database analysis established strong temporal consistency between the Web of Science and Scopus datasets *(r* = 0.985), with consistent bibliometric patterns across both databases, indicating a stable intellectual structure of the field.

**Conclusion:**

The findings suggest that communication is consistently conceptualized in the literature as a multilevel governance domain within patient safety systems. The proposed conceptual communication governance framework provides a structured basis for refining empirical data, developing clinical risk indicators, and conducting safety research involving nurses.

## 1 Introduction

Patient safety remains one of the key challenges of global healthcare, with medical errors regarded as a significant contributor to adverse clinical outcomes. The publication “To Err Is Human” was the first to systematically highlight the scale of preventable harm, establishing patient safety as a priority in clinical policy (1). Subsequent studies confirmed the high prevalence of adverse events in hospital care (2) and their importance for public health (3).

Recent studies show that approximately 6% of patients experience preventable harm, much of which is related to drug therapy and organizational factors (4). Despite progress, the risk profile is complicated by the development of outpatient care, an increase in diagnostic errors, and the digitization of healthcare (5, 6). According to the WHO, unsafe healthcare leads to millions of preventable harms each year, and the economic losses from medication errors are estimated at approximately US$42 billion (7).

Reason J. justifies a systemic approach to managing undesirable phenomena, contrasting it with an individualized model of error analysis (8). Within this paradigm, communication failures in healthcare, along with human, technological, and organizational factors, increase the risk of medical errors (9).

In the context of digital transformation, the implementation of electronic medical records, standardized protocols, and clinical decision support tools accelerates and optimizes information exchange and creates new opportunities for improving safety, while also introducing risks associated with data fragmentation and cognitive overload among staff (10).

Global health policy considers communication to be a key function of patient safety systems (11). The WHO strategy for 2021–2030 emphasizes transparency, interprofessional collaboration, and training as essential components of harm reduction, while the Patient Safety Rights Charter enshrines the patient’s right to timely and understandable information (12, 13).

Despite the growing number of publications on communication and medical errors, the evolution of this field remains fragmented. Most studies focus on specific clinical areas.

In the context of the rapid expansion of the scientific database, bibliometric analysis is becoming an important tool for systematizing and mapping research areas (14–16). For example, Konda et al. demonstrated exponential growth in publications on doctor-patient relationships, identifying dominant areas related to treatment adherence and clinical outcomes (17). Bataller-Alonso et al. systematized research on hospital communication, identifying key thematic areas, including doctor-patient communication, overcoming barriers, communication during transitions between institutions, and methodological approaches (18). Unlike previous bibliometric analyses that have focused separately on hospital communication or physician–patient interaction, this study integrates multiple communication domains within the broader patient safety literature, traces their thematic evolution, and synthesizes them into a governance-oriented conceptual framework.

In this regard, the following research questions (RQ) are formulated:

*RQ1*: How has the structure of research on communication and medical errors changed between 1964 and 2026?

*RQ2*: What is the role of communication in the patient safety knowledge network, and how has it changed over time?

*RQ3*: What communication governance themes and intervention areas are identifiable in the literature, and how can they be integrated into a conceptual framework?

This study undertakes a large-scale bibliometric analysis of the scientific literature connecting communication and medical errors, with the goal of proposing a conceptual communication governance framework that integrates the identified communication domains into a coherent structure pertinent to healthcare.

## 2 Methods

### 2.1 Research databases

The primary bibliometric dataset was extracted from the Web of Science Core Collection.

Each bibliographic database has distinct characteristics regarding the coverage and indexing of scientific literature (19, 20). In the present study, Web of Science was chosen as the primary data source because of its widespread application in bibliometric analyses, the availability of standardized bibliographic data, and its broad interdisciplinary coverage of the scientific literature.

The search for publications was conducted on 21 February 2026. This resource provides indexing of leading journals in the fields of clinical medicine, nursing, medical informatics, organizational sciences, psychology, and health care management, which is important for interdisciplinary analysis of communication issues and medical errors. In addition, Web of Science provides standardized bibliographic and citation data, as well as built-in analytical tools for studying citations, keywords, and scientific collaboration networks. To evaluate the consistency of the results, the Web of Science dataset was cross-validated using a complementary dataset from Scopus.

### 2.2 Search strategy

The search for publications was conducted using the fields “Title,” “Abstract,” and “Author’s keywords” without time restrictions. The following logical query was used for selection:

(“communication*” OR “miscommunication*” OR “information transfer*”) AND (“medical error*” OR “medication error*” OR “clinical error*” OR “diagnostic error*” OR “adverse event*” OR “adverse outcome*” OR “patient safety*” OR “patient harm*” OR “preventable harm*” OR “iatrogenic*”)

The OR operator broadened term coverage within each thematic group, while the AND operator ensured the selection of publications addressing both communication and clinical safety. The truncation symbol (*) allowed for the inclusion of different word forms.

The publication selection process followed the PRISMA framework adapted for bibliometric analysis (21) (Supplementary Figure S1). The initial search identified 13,544 publications; records were screened for duplication, and no duplicates were found. Retracted publications and corrections were excluded.

At the screening stage, publications were evaluated for compliance with the established relevance criteria, and materials classified under Veterinary Sciences and document types such as Book Chapter, Letter, and Note (*n* = 102) were excluded. A total of 13,431 publications were included in the final analysis.

The search strategy included English-language keywords, which is consistent with international bibliometric research practices. The sample also includes publications in other languages (German, Spanish, French, Portuguese, Korean, Italian, Japanese, etc.; *n* = 415). Most non-English publications had English titles and abstracts, which ensured their correct indexing and inclusion in the analysis. The linguistic structure of the sample reflects the global nature of research discourse, with English dominating.

### 2.3 Analytical tools

The initial descriptive analysis, including annual publication and citation dynamics, distribution by country, organization, publisher, journal, and Web of Science category, was performed using the built-in tools of the Web of Science platform, including the Analyze Results and Citation Reports functions.

Percentages by country/regions and institution reflect non-exclusive attribution in Web of Science records, where multi-author publications may be assigned to multiple affiliations.

An extended bibliometric analysis was conducted in R (version 4.5.2; R Core Team, 2025) using the bibliometrix package (22) for metadata extraction and structuring, productivity assessment, and thematic evolution analysis. The Trend Topics function was used to study the temporal dynamics of keywords, including peak bursts of mentions reflecting periods of increased scientific interest; the analysis included only terms with a frequency of ≥5, which allowed low-frequency words to be excluded and improved the interpretability of the maps (22).

Visualization of keyword and international collaboration networks (1964–2026) was carried out using VOSviewer (version 1.6.18) (23). For the keyword analysis, only keywords with a frequency of ≥0.45% of the total publications (≥60) were included, enhancing the clarity and interpretability of thematic clusters (23).

### 2.4 Justification for temporal periodization

The temporal segmentation of the analysis (1964–1999; 2000–2010; 2011–2019; 2020–2026) was determined based on the annual dynamics of publication activity and the thematic evolution of the research field, using the Web of Science and bibliometrix tools. The analysis revealed three distinct inflection points: the transition from low publication density (<15 publications per year) to steady growth after 2000; the acceleration of publication activity after 2011; and the emergence of a new scale after 2020, exceeding 900 publications per year (the 2026 figures were interpreted taking into account incomplete indexing). These changes reflect the non-linear nature of the development of the scientific field and justify the identification of four analytical phases.

The period 1964–1999 was characterized by low publication density and a clinical-descriptive model of analysis, within which communication was not considered a systemic risk factor. From 2000 onward, following the publication of “To Err Is Human,” there was an exponential increase in publication activity and broad recognition of the patient safety concept (2000–2010), accompanied by a rising frequency of keywords such as patient safety, systems, risk management, and communication.

During 2011–2019, thematic differentiation intensified, with increased focus on patient safety, the prevention of adverse events and adverse drug events, improvement of communication and teamwork (e.g., crew resource management, briefings), as well as evaluation of care quality (care, quality improvement). From 2020 onward, research has increasingly shifted toward the digital transformation of healthcare (artificial intelligence), while continuing to emphasize patient safety, communication, teamwork, and error prevention in healthcare systems.

Thus, the proposed segmentation reflects the evolution of research focuses: from a clinical-descriptive model to a systemic safety paradigm and further to the digital transformation of communication in healthcare.

It is worth noting that the references to artificial intelligence and ChatGPT in the results reflect thematic trends identified in the analyzed scientific literature.

### 2.5 Data validation and cross-database approach

To enhance the robustness of the results, the primary dataset retrieved from the Web of Science Core Collection was cross-validated against a complementary dataset extracted from Scopus (1964–2026) using the same search strategy and keywords. The validation procedure included both quantitative and qualitative components. Quantitative validation involved comparing publication output dynamics and calculating Pearson’s correlation coefficient to assess temporal consistency between the two databases. Qualitative validation included the comparison of highly cited publications and keyword bursts across the four research stages using bibliometrix. In addition, to assess the stability of the intellectual structure, the composition of keyword clusters identified using VOSviewer was compared between the Web of Science and Scopus datasets.

## 3 Results

### 3.1 General bibliometric characteristics

As a result of the literature search, a database of 13,431 documents devoted to the study of communication and medical errors was compiled, covering the period from 1964 to 2026. This enabled the analysis of long-term trends in scientific activity and the dynamics of publications, distribution by document type, subject category, country, leading organizations, journals, and publishers.

**FIGURE 1:**
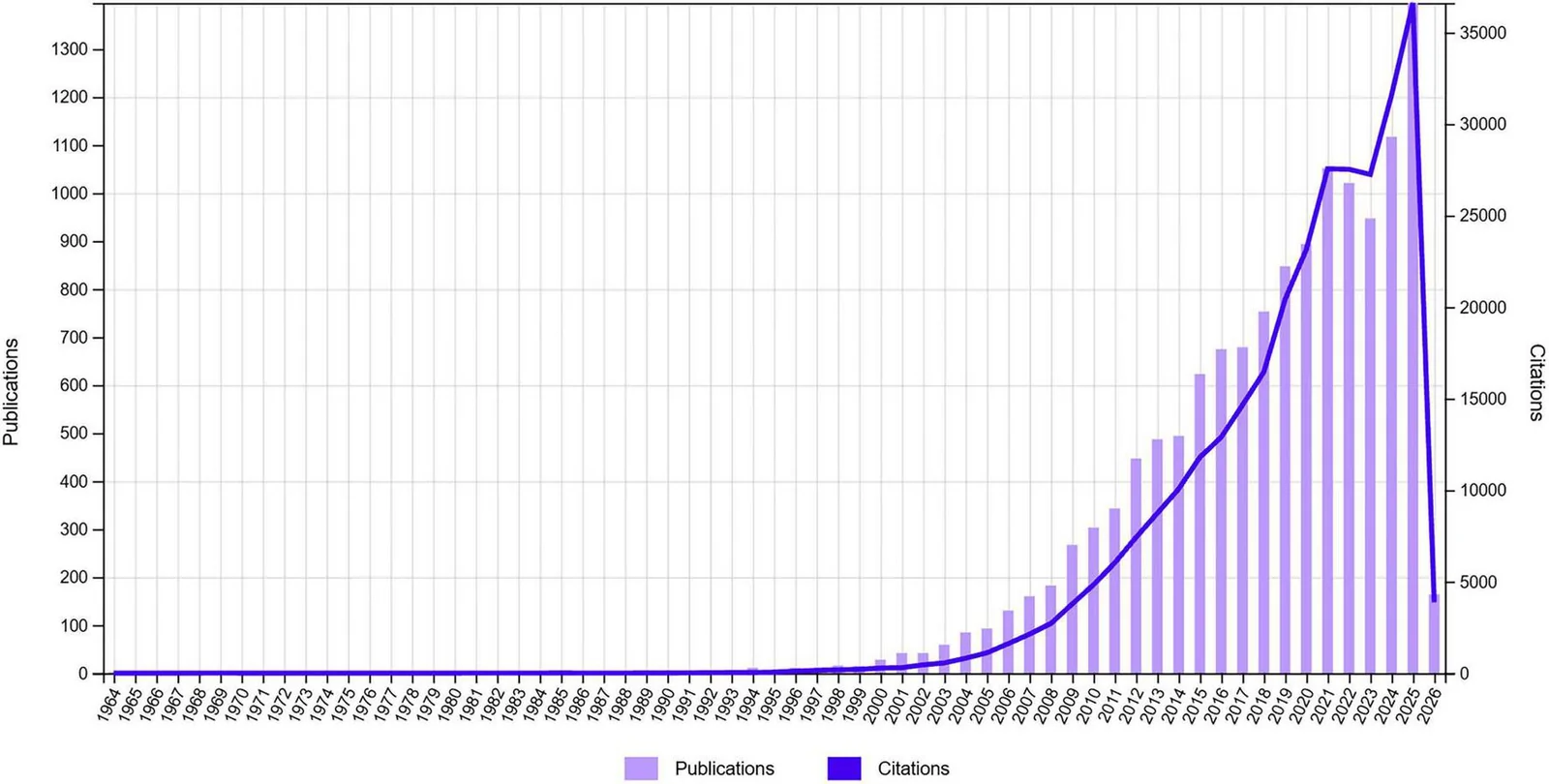
Dynamics of publications and citations by year in the field of communication and medical errors (1964–2026); Due to the timepoint (21 February 2026) of data extraction, publications in 2026 are fewer in number and have not yet had sufficient time to accumulate citations, which is reflected in their currently lower citation counts.

An analysis of publication activity showed that in the early years, research on the topic was extremely limited: from 1964 to 1990, the number of publications rarely exceeded between 5 and 10 per year. Since 1991, there has been a gradual increase in interest in the topic, reflecting the medical community’s growing awareness of the importance of communication for patient safety. Between 2000 and 2010, the number of publications increased to 28–303 per year, and since 2010, there has been steady and rapid growth. The highest productivity was recorded in 2025 (1,399 publications) and 2024 (1,117 publications). As of 2026, 168 publications have been registered, indicating continued interest in researching communication as a key factor in preventing medical errors (Figure 1). It is worth noting that the 2026 data are incomplete, as data collection was completed on February 21, 2026. Citation counts are reported by publication year and are cumulative; articles published in 2026 show lower citation numbers due to the timepoint of data collection (21 February 2026), and limited time to accumulate citations.

An analysis of document types showed that most publications are articles (80.3%). A significant proportion of reviews (13.7%) indicates the systematization of accumulated knowledge. Conference materials (4.2%) and other forms of publications (editorial materials, etc.) account for less than 5% of the total number, which emphasizes the predominance of original research and scientific reviews in this field.

Thematic analysis revealed that research in the field of communication and medical errors exhibits a strongly interdisciplinary character. The most represented categories include health care and the organization of medical services (20.7%), nursing (13.5%), and general internal medicine (13.3%). A significant number of publications are also related to health policy (9.8%) and public health (7.1%). These data reflect a variety of scientific approaches: from the analysis of organizational processes and professional communication to the prevention of errors at the level of healthcare systems. Other significant categories include surgery, pharmacology, medical informatics, pediatrics, and education, demonstrating the widespread prevalence of the topic in various fields of medicine and science (Supplementary Figure S2).

Geographical analysis showed that research in the field of communication and medical errors is actively conducted in countries/regions with developed healthcare systems and scientific infrastructure. The largest number of publications comes from the United States (17.4%), England (5.2%), Australia (4.2%), Canada (3.2%), and Germany (2.7%). The top 10 also includes China, Spain, Italy, Switzerland, and India, each with a share ranging from 1.3 to 2.5%. Together, these countries/regions account for about 41% of all publications, demonstrating the concentration of scientific activity in the world’s leading centers of medicine and patient safety (Supplementary Table S1).

**FIGURE 2:**
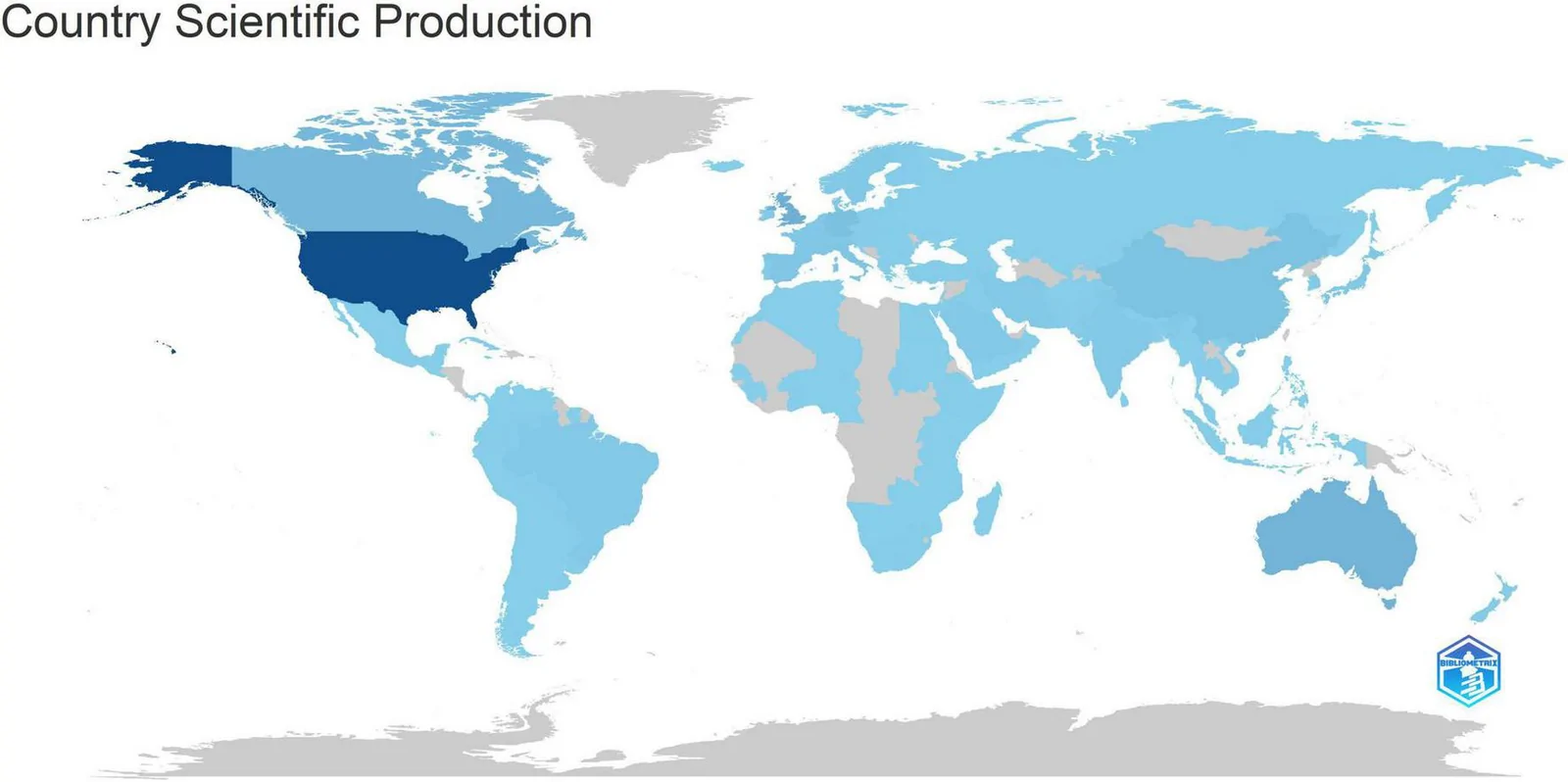
Global productivity of research in the field of communication and medical errors (as of 21 February 2026).

The global productivity of research on communication and medical errors covers 139 countries/regions, demonstrating comprehensive international interest in the issue and a broad geographical distribution of scientific activity; the corresponding visualization is presented on the world map in Figure 2.

Among publishers, Elsevier (7.8%), Springer Nature (5.2%), and Wiley (5.0%) show the highest productivity, reflecting their key role in disseminating knowledge about communication and medical errors. The leading journals are the Journal of Patient Safety (1.9%), BMC Health Services Research (1.6%), and BMJ Quality and Safety (1.5%), which indicates the strong interest of the professional community in the topic of patient safety through effective communication. Among organizations, the largest contributions came from Harvard University (5.5%), Harvard University Medical Affiliates (4.9%), the University of California System (3.3%), Harvard Medical School (3.2%), and the University of Toronto (2.7%). These data underscore the importance of leading academic and clinical centers in promoting research aimed at reducing medical errors through improved communication (Supplementary Table S2).

Thus, the results of the bibliometric analysis demonstrate steady growth in publication activity in the field of communication and medical errors, widespread coverage of the topic in various disciplines, concentration of research in leading countries and organizations, and the dominant role of major publishers and specialized journals in the dissemination of knowledge.

### 3.2 The evolution of research in the field of communication and medical errors (1964–2026)

A bibliometric analysis using the bibliometrix package identified four successive phases in the development of this field of research, differing in terms of publication activity, keyword structure, and publication topics.

#### 3.2.1 Stage I (1964–1999) clinical description of adverse events and early problematization of communication

The first stage is characterized by low publication activity and a predominance of clinical descriptive studies. Despite the formal start of the stage in 1964, a steady increase in the frequency of keywords has only been recorded since 1994, reflecting the later formation of the thematic focus of research. The most frequent keywords (adverse events, care, hospitalized patients, anesthesia, and communication) reflect the focus on recording complications and adverse outcomes, mainly in inpatient practice (Supplementary Figure S3). Communication is not a central category during this period and is considered only episodically.

At this stage, highly cited publications form the basis for understanding the role of communication in medical practice. For example, Wu showed that discussing mistakes with colleagues and patients is associated with professional changes, emphasizing the importance of open communication (24). Donchin et al. identified a link between human errors in intensive care units and problems with interprofessional communication, including between doctors and nurses (25). Beckman et al. demonstrated that the causes of lawsuits are often related to insufficient communication and disregard for the patient (26). Brown et al., analyzing the implementation of the Medical Dictionary for Regulatory Activities, focused on the standardization of terminology and the quality of data transfer in pharmacovigilance systems (27).

Thus, despite the predominance of clinical documentation of adverse events, by the end of the period, there is empirical evidence that communication breakdowns are associated with both clinical and legal consequences of medical practice.

#### 3.2.2 Stage II (2000–2010) communication and medical errors in the patient safety system

During this period, research into communication and medical errors was considered in the context of the patient safety system, where errors were interpreted as the result of communication breakdowns, insufficient coordination, and organizational failures. The most frequently occurring keywords (patient safety, communication, adverse events, error, care, adverse drug events, systems, risk management) reflect the formation of a systematic approach focused on preventing adverse outcomes (Supplementary Figure S4).

During this period, highly cited publications emphasized the importance of communication in preventing errors. For example, Kripalani et al. showed that a lack of information transfer when discharging patients creates conditions for therapeutic and medication errors (28). Coleman et al. demonstrated that structured support for patients during transitional periods reduces the risk of readmissions and adverse events (29). Leonard found that most serious adverse outcomes are associated with communication breakdowns and the lack of standardized tools for information transfer (30). Ash et al. described that the implementation of clinical information systems may inadvertently contribute to medical errors, including through disruptions in communication and coordination processes (31).

Particular attention was paid to the side effects of drugs. Thus, the recommendations of Ansell et al. emphasize the need for systematic monitoring, patient education and coordinated information transfer during therapy with vitamin K antagonists to prevent bleeding and thrombotic complications (32).

Accordingly, the period from 2000 to 2010 marked the emergence of a systemic patient safety model. Errors were increasingly understood as consequences of organizational and informational failures, and prevention strategies emphasized effective communication, standardized processes, and risk management.

#### 3.2.3 Stage III (2011–2019) patient-centered communication and medical errors: the role of the team and the human factor

In 2011–2019, research shifted toward team communication, the human factor, and patient-centered interaction as key mechanisms for preventing medical errors. The most frequently occurring keywords (patient safety, communication, care, adverse events, errors, quality improvement, crew resource management, briefings, surgical safety, and emergency department) reflect the growing attention to team coordination, high-risk departments, and improving organizational effectiveness (Supplementary Figure S5).

Highly cited publications from 2011 to 2019 highlight the impact of communication on errors and patient safety. For example, Reeves et al. showed that interprofessional educational interventions improved teamwork, reduced the frequency of clinical errors, and increased professional competence (33). Doyle et al. demonstrated that involving patients in discussions about treatment plans improves safety and reduces the risk of adverse outcomes (34). It has been reported that the use of decision-making techniques increases patient awareness of risks and reduces the likelihood of therapeutic errors (35).

Regular use of questionnaires to assess treatment outcomes reported by patients enhances feedback; helps identify errors in care and adjust treatment, improving compliance and patient satisfaction (36).

Thus, the period from 2011 to 2019 is characterized by the formation of a systematic, patient-centered approach in which medical errors are viewed as the result of team communication breakdowns, human factors, and interactions between patients, relatives, and the medical team.

#### 3.2.4 Stage IV (2020–2026) digital transformation and hybrid, omnichannel communication models

**FIGURE 3:**
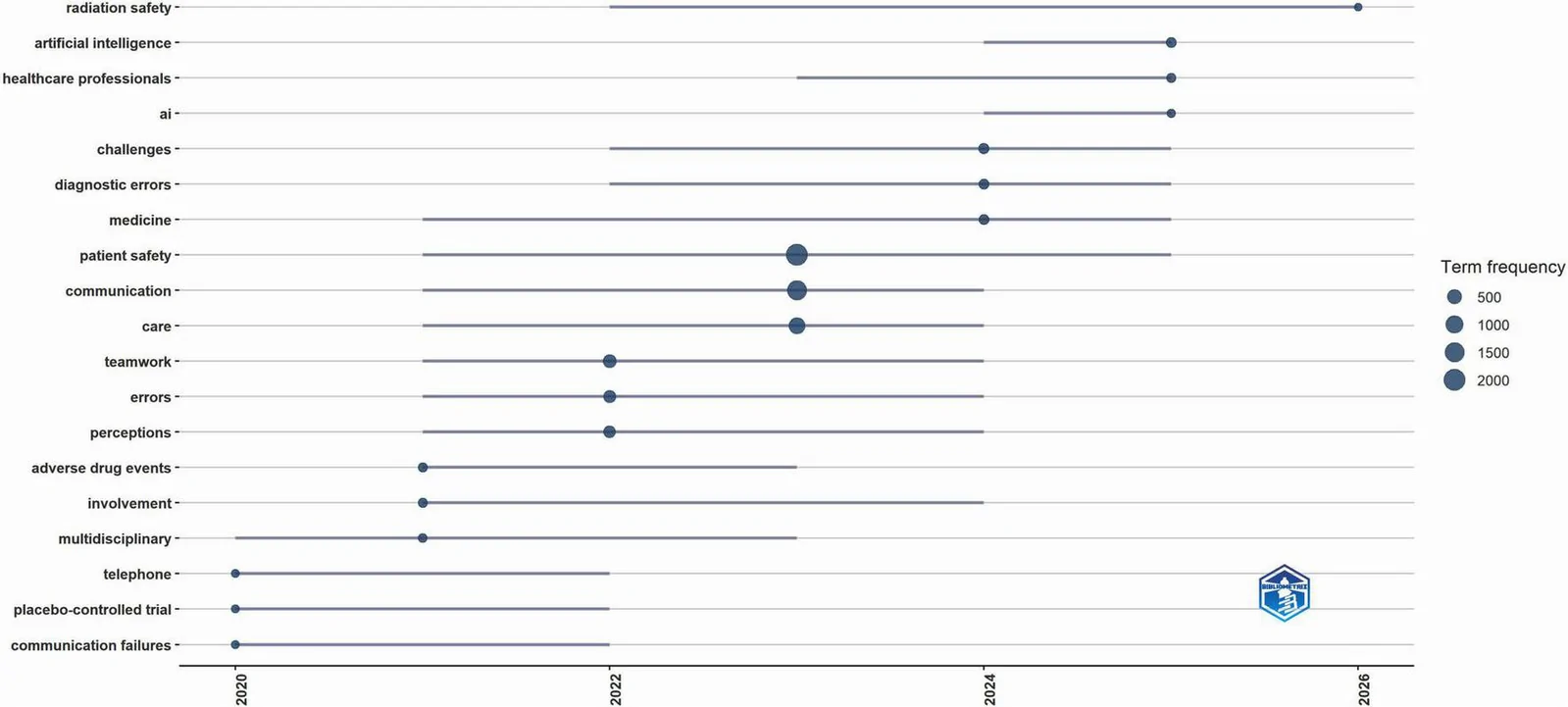
Keyword trends with peak bursts ( ≥5), bibliometrix, 2020–2026.

In 2020–2026, analysis shows an increase in keywords related to communication, teamwork, and quality of care (patient safety, communication, errors, teamwork, perceptions), as well as digital transformation (artificial intelligence, diagnostic errors, telephone, multidisciplinary) (Figure 3).

Highly cited publications from this period highlight the considerable future potential of large language models, such as ChatGPT, in medical practice, research, and training, but which carry the risk of misinformation (37).

At the same time, research on team effectiveness confirms that training based on the principles of CRM (crew resource management) and TeamSTEPPS (Team Strategies and Tools to Enhance Performance and Patient Safety), as well as simulation-based training, effectively improves teamwork and reduces the number of errors (38).

The COVID-19 pandemic has increased the dependence of clinical processes on remote channels, revealing new types of communication gaps. Telemedicine and electronic documentation systems have become key intermediaries for information transfer, and attention to diagnostic errors has increased due to cognitive overload and data fragmentation. A new type of interaction is emerging—“doctor-algorithm”—where information systems become active participants in the communication process, creating a hybrid, omnichannel model that combines face-to-face visits, remote consultations, electronic records, and digital patient platforms (39).

### 3.3 Thematic clusters as a reflection of knowledge structure

**FIGURE 4:**
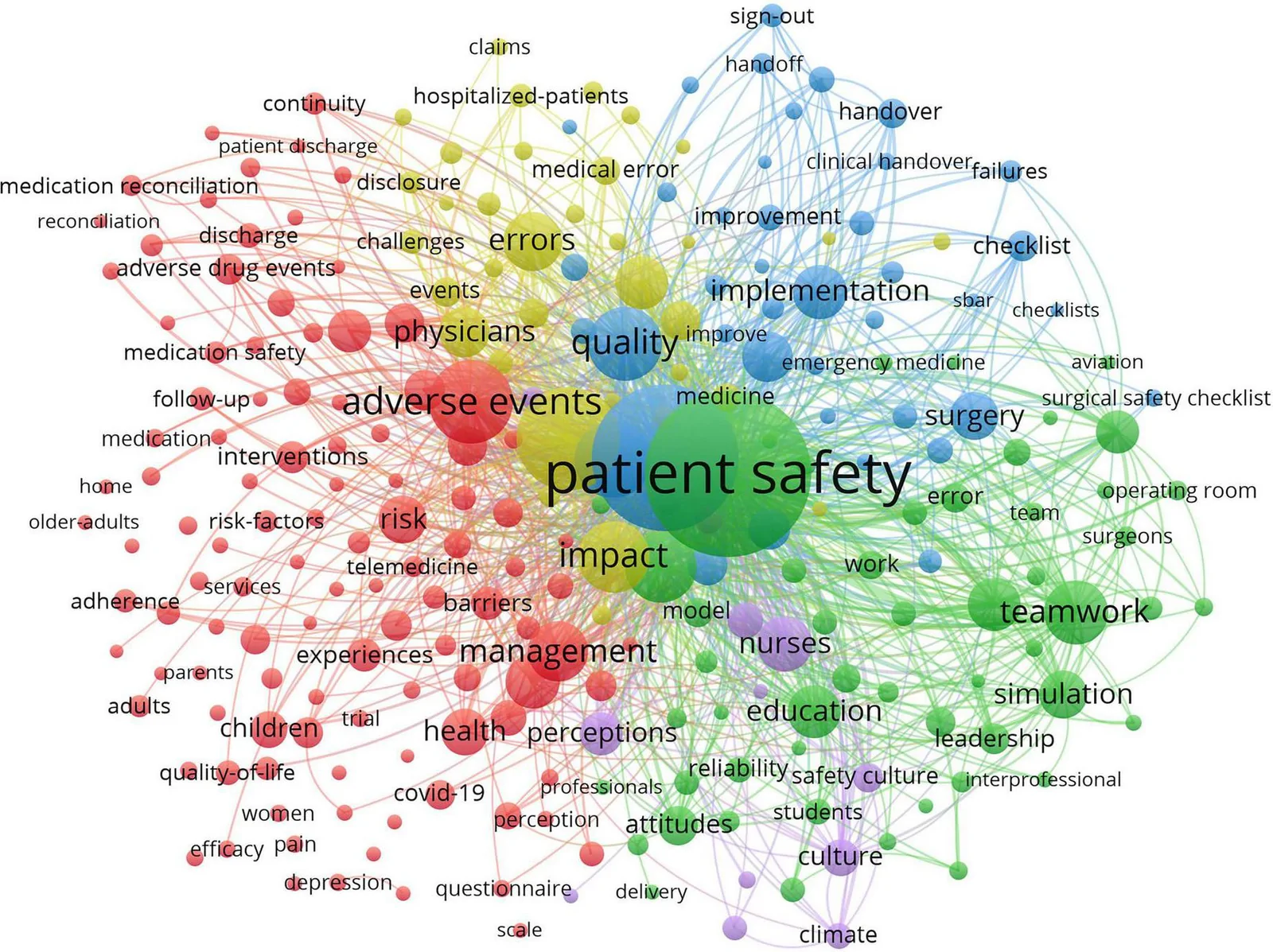
Map of keywords and thematic clusters of publications on communication and medical errors (Web of Science) 1964–2026.

Visualization of keywords using VOSviewer revealed the structure of knowledge in the field of communication and medical errors for the period 1964–2026. The size of the circles on the map reflects the frequency of use of terms, and their proximity reflects the strength of the relationships, allowing us to trace both the internal connections within individual topics and the interaction between different areas of research. The analysis revealed five thematic clusters, each of which reflects a separate research area and demonstrates the characteristics of communication and medical errors in clinical practice (Figure 4).

The red cluster is dedicated to pharmacotherapy safety and clinical incidents. The main focus is on the processes of transferring information about therapy, coordinating prescriptions, maintaining documentation, and preventing errors in the use of medicines. Communication in this area takes place between doctors, pharmacists, and patients, as well as through documentation and pharmacovigilance, reflecting the complex interaction of all participants in the clinical process. Research in this cluster is aimed at identifying and preventing medical errors and adverse events (adverse drug events, pharmacovigilance, medication errors, medication safety, documentation, telemedicine, malpractice claims).

The green cluster reflects teamwork and human factors. The main focus is on interprofessional coordination, developing teamwork skills, leadership, training, and ensuring psychological safety. Communication is a key mechanism for interaction between doctors, nurses, and other team members, as well as for organizing teamwork. These studies aim to reduce errors in clinical practice and prevent adverse events through effective teamwork (teamwork, non-technical skills, simulation, leadership, education, psychological safety, errors, and adverse events).

The blue cluster focuses on information transfer and patient-centered communication. It covers structured clinical information transfer processes, the use of checklists and handover standards (standardized transfer of patient information from one healthcare professional or team to another during shift changes, patient transfers or transfers of responsibility for treatment), as well as strategies for improving patient safety and quality of care. Of particular importance is the interaction of healthcare professionals with patients in intensive care and emergency medicine departments, which helps to reduce errors and improve the quality of care (handover, handoffs, patient handoff, checklist, SBAR, safety, quality improvement, complications, morbidity, mortality, patient care, errors, and adverse events).

The yellow cluster brings together research on digital infrastructure and hybrid, omnichannel communication. It reflects the introduction of electronic medical records, artificial intelligence, and information technology into clinical practice, creating new forms of interaction between doctors and AI, patients and digital platforms, and between organizations. These technologies help reduce diagnostic and medical errors, improve the accuracy of clinical information, and the quality of care (electronic health records, artificial intelligence, information technology, diagnostic errors, and medical errors). At the same time, they carry potential risks.

The lilac cluster focuses on organizational culture and risk management, viewing communication as a systemic mechanism for ensuring patient safety. Nursing practice plays a particularly important role in this structure. The results of the analysis show that nurses are key coordinators of information flows in the healthcare system. The processes they initiate, including medication administration, patient education, and interprofessional coordination, are critical nodes where communication failures can either increase or decrease the risk of errors. Communication in this context is understood as a management function that ensures process transparency and maintains a culture of safety. Thus, the cluster emphasizes the role of effective management and nursing leadership in preventing medical errors (safety culture, organizational culture, risk management, medical errors, adverse events, nurses).

### 3.4 Interaction between research communities

Network analysis revealed a heterogeneous structure of international cooperation distributed across several macro-regional clusters (Supplementary Figure S6). A broad co-authorship node brings together countries from the Middle East, South Asia, and Southeast Asia, forming a stable regional vector of publication activity. In parallel, a European integration network was identified, linking research centers in Central and Eastern Europe with the Israeli research sector. The central research segment is characterized by close interaction between several European countries (Germany, France, Switzerland, Scandinavia) and key scientific and technological hubs in East Asia. Simultaneously, the English-language cluster demonstrates high internal connectivity between the United Kingdom, Australia, and New Zealand. Interregional interaction is further evidenced by the integration of Latin America with Spain, as well as the links between African research communities and the United States academic ecosystem.

These results highlight the existence of robust global collaboration networks that enable the identification of key centers of scientific activity and reflect the importance of international cooperation for the advancement of research in the field of communication and medical errors.

### 3.5 Validation of bibliometric trends and cross-database consistency

#### 3.5.1 Cross-database validation of publication counts

To assess dataset robustness, publication counts obtained from Web of Science were compared with those retrieved from Scopus across four time periods (1964–2026). The results are summarized in Table 1.

**TABLE 1:** Cross-database validation of publication counts by stage in Scopus and Web of Science (1964–2026).

Stage	Scopus	Web of Science	Ratio (Scopus/Web of Science)
1964–1999	1,214	102	11.9
2000–2010	4,996	1,390	3.6
2011–2019	10,904	5,348	2.0
2020–2026	11,738	6,591	1.8

While Scopus yielded a higher number of records due to its broader source coverage, the temporal evolution of publications was highly consistent between the two databases. The Pearson correlation coefficient across the analyzed periods reached 0.985, demonstrating strong agreement in publication trends and supporting the reliability of the Web of Science dataset for subsequent analysis.

#### 3.5.2 Qualitative validation using highly cited articles

Additionally, a qualitative validation was conducted based on highly cited publications across different research stages. For each stage, the most cited documents in Scopus and Web of Science were compared to assess overlaps and thematic consistency. The analysis revealed partial but stable correspondence of highly cited articles between the databases, for example: Wu A.W. (1991) (24), Beckman H.B. (1994) (26), Donchin Y. (1995) (25), Brown E.G. (1999) (27), Leonard M. (2004) (30), Ash J.S. (2004) (31), Kripalani S. (2007) (28), Ansell J. (2008) (32), Stacey D. (2017) (35), and Buljac-Samardzic M. (2020) (38).

#### 3.5.3 Validation of keyword trends with peak bursts using Scopus

To validate the findings, a comparative analysis of keyword bursts was conducted across the Web of Science and Scopus databases, aligned with the identified research stages. The results demonstrate high cross-database consistency. Despite quantitative differences in the samples, the structure of key themes and their evolutionary dynamics remain comparable. In stage I (1964–1999), Web of Science and Scopus equally record a dominance of clinical issues related to adverse outcomes and medical errors (adverse events, iatrogenic disease, diagnostic errors), alongside the emerging role of communication components. In stage II (2000–2010), a synchronous shift toward a systems approach to patient safety is observed in both databases, reflected in the growth of terms such as patient safety, medical error, and communication, as well as categories of risk management and process standardization (risk management, checklist). In stage III (2011–2019), both databases indicate a transition to patient- and team-oriented models, accompanied by an increase in terms related to education and performance enhancement (simulation, training, quality improvement). In stage IV (2020–2026), Web of Science and Scopus demonstrate a shared trend toward digital transformation, with an intensifying focus on artificial intelligence, telemedicine, and teamwork. The high degree of keyword overlap and the synchronicity of their emergence confirm the robustness of the identified intellectual structure, proving it to be independent of the specific bibliographic source.

#### 3.5.4 Validation of thematic clusters using Scopus

To validate the thematic clusters identified in the Web of Science dataset, a cross-database comparison was conducted using Scopus. Given the larger size of the Scopus dataset (28,852 publications), the analysis was restricted to keywords with a frequency ≥ 300 to ensure that the resulting clusters reflected the most significant and frequently discussed topics.

Each cluster identified in Web of Science was mapped to corresponding keywords in Scopus to assess the consistency of research directions. The analysis demonstrated a high degree of concordance between the two databases: the principal research areas—pharmacotherapy and medication safety, teamwork and human factors, information transfer and patient-centered communication, digital infrastructure and technology, and organizational culture and risk management—were consistently represented across both datasets. While Scopus exhibited slightly broader topical coverage, reflecting its wider journal indexing, the core thematic clusters remained stable. These findings confirm that the clusters derived from Web of Science robustly capture the underlying thematic structure of the field.

Comparative analysis of Scopus and Web of Science data demonstrates a high level of consistency across both quantitative and qualitative levels of analysis. Despite differences in publication volumes, both databases reveal similar patterns in the development of the research field, alignment in key thematic shifts, and a stable cluster structure. These findings confirm the reproducibility and robustness of the identified bibliometric trends, as well as the reliability of the results based on the Web of Science dataset.

## 4 Discussion

The bibliometric analysis indicates not only quantitative growth but also a gradual conceptual transformation of the scientific discourse. While early studies primarily documented adverse events as individual clinical errors, contemporary literature increasingly frames medical errors as associated with multi-level systemic interactions, within which communication appears prominently positioned.

The findings of the present study are consistent with recent bibliometric analyses of healthcare communication, which have identified patient–physician communication, interprofessional collaboration, patient safety, communication skills, methodologies, and transitions of care as major research themes (17, 18). Similar thematic areas were identified in the present analysis, indicating sustained scholarly interest in these topics. However, whereas previous bibliometric studies have largely focused on specific aspects of communication, the present study examines communication within the broader context of medical errors and patient safety. Based on the bibliometric findings, a conceptual five-level communication governance framework was proposed, synthesizing the identified research domains into a coherent whole. It should also be acknowledged that differences among bibliometric studies may be influenced by variations in the databases used, search strategies, time periods analyzed, and research focus scope, which may lead to alternative interpretations of the observed patterns.

The identified developmental phases (clinical-descriptive, systemic, team- and patient-centered, and digital) reflect an expansion of analytical attention, from isolated clinical acts to complex networked interactions. At the present stage, communication is increasingly described as hybrid, omnichannel, and technologically mediated, forming evolving interaction patterns such as “doctor–patient,” “team–team,” and “doctor–algorithm–patient.”

The interdisciplinary character of the field is supported by the dominance of Health Care Sciences Services, Nursing, and General Internal Medicine categories, as well as increasing representation in health policy and public health. This distribution suggests a broadening from micro-level clinical risk management toward macro-level clinical risk governance perspectives. The concentration of scientific output in countries/regions with developed research infrastructures reflects structural research capacity.

The identified stages and cluster structures demonstrate parallel thematic developments. Together, they reflect a coordinated expansion of the analytical scope of the field. Across thematic clusters, communication emerges as an important element within the patient safety knowledge network. It performs an integrative function, ensuring cognitive, organizational, and technological consistency of clinical processes. Disruptions in any segment of this network can generate clinical risk.

To ensure the reliability of the findings, we performed a cross-database validation procedure. Comparing Web of Science and Scopus data across the analyzed stages established a high Pearson correlation coefficient (*r* = 0.985), along with consistent qualitative patterns. This level of alignment indicates that the identified structural transitions in the scientific discourse are robust and consistently reflected across different authoritative scientometric databases.

The pharmacotherapy safety cluster suggests that medication-related harm is frequently discussed in connection with information transfer, documentation, and coordination processes (40). Communication here ensures the synchronization of actions between doctors, pharmacists, nurses, and patients. At the macro level, international drug side effect monitoring systems (VigiBase, EudraVigilance) enhance data systematization and safety control (41). The cluster’s findings suggest that a significant proportion of medical errors occur as a result of breaks in communication chains, which is consistent with Reason J. systemic logic (8). Thus, the pharmacotherapeutic cluster indicates that some preventable harm is associated not only with drug side effects but also with disruptions in communication flows.

Similarly, the teamwork and human factors cluster highlights literature that associates interprofessional communication, psychological safety, and structured handover protocols with improved safety outcomes (42, 43). Standardized information transfer (44), team training, and interprofessional training (45, 46) are associated with a reduction in diagnostic and clinical errors. Barriers related to hierarchy and role fragmentation (47, 48) directly affect the risk of errors, and interactive training formats demonstrate high effectiveness (49). These findings align with existing systemic safety theories. The cluster informs that errors can reflect systemic communication failures even in the presence of clinical competence.

The patient-centered cluster reflects literature linking communication quality with adherence, diagnostic clarity, and patient engagement. Here, communication is portrayed as a relational and interpretative mechanism shaping care processes (50).

Thus, the PC4 model for nurses and patients demonstrates that its effective implementation at the highest level depends on developed communication skills, organizational support, sufficient time, and cultural competence, which contribute to reducing the frequency of errors and improving clinical outcomes (51). Language (52), cultural (53), racial (54), and social (55) barriers increase the risk of adverse events and distort the dynamics of the doctor-patient interaction. Empathetic (56) and structured communication (57), family involvement (58), and the use of digital tools (59) improve adherence to recommendations and prevent errors, especially in chronic (60), stigmatized (61), oncological (62) diseases and at the end of life (63). Empirical data show that an active stance on the part of patients and relatives can “save the day” and prevent errors, while ignoring their signals is associated with adverse outcomes (64). Patients and relatives become active participants in the communication ecosystem.

Digital transformation cluster positions communication as increasingly mediated by technological infrastructures, including a hybrid, omnichannel communication model (65), where interaction between participants is mediated by telemedicine (66), mobile applications (67), online platforms, social networks (68), Chatbots (69), digital biosensors (70), and AI. New forms of interaction, “doctor-algorithm-patient,” increase the accuracy, coordination, and personalization of clinical decisions. Digital ecosystems enhance care coordination, support shared decision-making, increase medical literacy and patient engagement, and facilitate personalized communication and optimized clinical decisions (65).

Research confirms that integrating AI into clinical practice opens up opportunities to improve diagnostic accuracy, personalize treatment, optimize drug dosages, support decision-making, and reduce human error (71, 72).

Educational digital formats (73), virtual patients, simulations (74, 75) and AI-assisted training (76) indicate comparable effectiveness to traditional training in developing communication skills and allow complex clinical situations to be safely modeled.

However, the implementation of digital technologies is associated with risks: misinformation (77), threats to data confidentiality, algorithmic bias, information overload, and technological failures (78). The bibliometric structure captures this thematic duality. To minimize these risks, data verification, compliance with ethical standards, and adaptation of digital solutions to clinical practice are necessary.

The organizational culture cluster emphasizes leadership, transparency, and structured reporting systems. Within this body of literature, communication is conceptualized as a governance-related process contributing to safety culture formation. Leadership, process standardization, the implementation of structured models (SBAR, Situation, Background, Assessment, and Recommendation) (79) and transparent discussion of errors strengthen the safety culture, increase staff satisfaction and patient trust (80). The organizational climate and management support directly influence the willingness of employees to “speak up,” which reduces the likelihood of repeat incidents (81).

These findings are particularly relevant for nursing practice. Nurses, positioned at the center of clinical processes, ensure continuity of communication, coordination of interprofessional collaboration, responsibility transfer, and documentation of clinical data. Interpreting medical errors as a breakdown in the communication loop emphasizes the strategic role of nurses in maintaining its stability. Strengthening and enhancing nurses’ leadership competencies, developing structured information transfer skills, and fostering a culture of open error discussion represent key directions for improving patient safety.

Importantly, medical errors cannot be attributed exclusively to communication factors. Clinical uncertainty, cognitive biases, resource limitations, and technical failures represent additional interacting determinants. Nevertheless, keyword co-occurrence patterns indicate a structurally central positioning of communication within the research network. Information flows either compensate for potential risks or amplify and spread them. Consequently, communication is not the sole cause of errors, but it is a critical mechanism for their systemic transmission.

**FIGURE 5:**
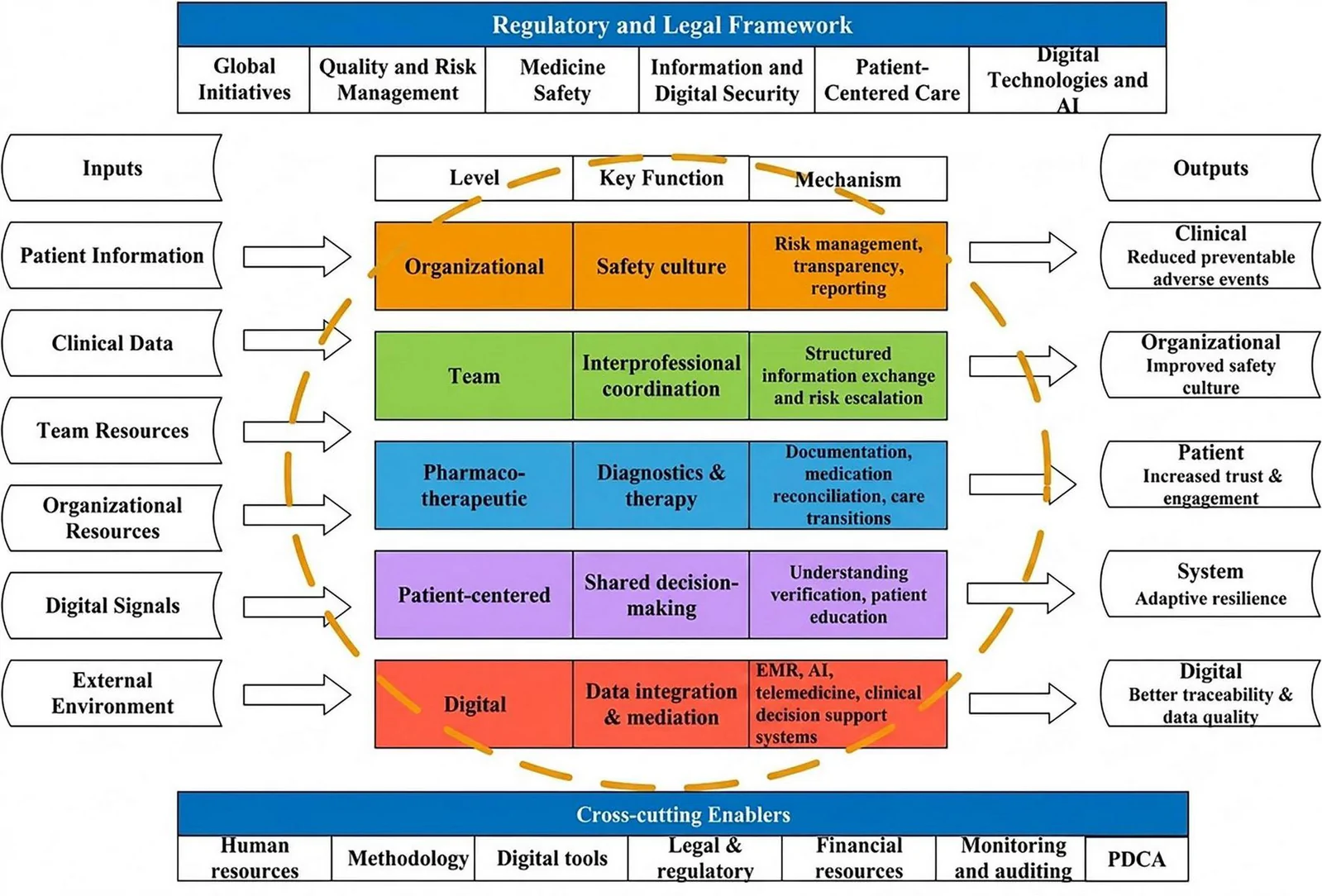
Conceptual communication governance framework.

A five-level conceptual communication governance framework is proposed as a post-bibliometric synthesis grounded in recurring thematic configurations (Figure 5). The framework organizes communication-related domains identified in the literature into an analytically coherent structure and provides a foundation for future empirical research:

Organizational level: shapes a culture of safety, standardizes information transfer processes, ensures transparency of reporting, and psychological safety of staff.

Team level: coordinates interprofessional work, supports briefings and feedback mechanisms, reducing errors in the transfer of responsibility.

Pharmacotherapeutic level: responsible for documentation, therapy coordination, and critical information transfer, preventing diagnostic and medical errors.

Patient-centered level: involves the patient and family through information sharing, joint decision-making, and monitoring of understanding of recommendations.

Digital level: mediates the interaction between the clinician, the system, and the patient, improves the accuracy and traceability of information, while requiring control of information overload and cyber risks.

The model operates through the PDCA cycle: Plan—identification of communication risks, Do—implementation of protocols, Check—monitoring of incidents, Act—adjustment of processes. Errors occur when there is a breakdown in the transmission of information, distortion of meaning, inconsistency in decisions, fragmentation of data, or disruption of feedback.

For healthcare management, including nursing practice, the framework offers a structured lens for examining communication reliability as a nursing-sensitive safety dimension. Nurses operate across organizational, team, pharmacotherapeutic, and patient-centered interfaces; therefore, strengthening communication governance may represent a modifiable system-level pathway for safety improvement.

Overall, the findings suggest that communication occupies a structurally central and integrative position in patient safety research, while acknowledging that bibliometric associations do not constitute empirical verification of causality.

This study contributes to patient safety research by providing a bibliometrically informed conceptual synthesis of communication-related domains within the medical error literature.

This study advances patient safety theory by reconceptualizing medical error not as an isolated clinical event but as a manifestation of disrupted communication integrity across interconnected system levels.

The proposed framework integrates organizational, team, pharmacotherapeutic, patient-centered, and digital themes into a structured analytical schema derived from bibliometric patterns. It offers a conceptual organization of recurring thematic configurations identified over six decades of research.

By structuring these domains within a communication governance perspective, the model reorients attention toward communication reliability as a systemic dimension of patient safety, particularly in increasingly digital and hybrid healthcare environments.

The findings of the present study may have implications for healthcare professions education, as they highlight the multilevel nature of communication within contemporary healthcare systems. In particular, the identification of organizational, team, pharmacotherapeutic, patient-centered, and digital communication levels suggests the need for further exploration of how these dimensions can be incorporated into the education and training of healthcare professionals and interprofessional education programs. Furthermore, the emergence of a digital communication governance level reflects the growing role of technology in healthcare and underscores the importance of research aimed at preparing healthcare professionals for the evolving physician–algorithm–patient model of interaction. In this context, particular attention may need to be given to developing competencies related to the critical interpretation of algorithm-generated recommendations, effective interaction with digital decision-support systems, and the maintenance of a patient-centered approach when applying technology in clinical practice.

## 5 Limitations

This study has several limitations. First, the analysis was based on Web of Science data with cross-validation using Scopus; accordingly, some publications indexed outside these databases may not be reflected in the analysis. Second, the search strategy used English-language keywords, which potentially limits the coverage of studies without English-language metadata. Consequently, studies published in languages other than English that lacked English-language abstracts, as well as publications in local and regional journals, may have been underrepresented in the analysis. Third, the bibliometric approach reflects the structure of published scientific discourse but does not allow for an assessment of the methodological quality of individual studies or the clinical effectiveness of communication interventions. Therefore, the prevalence of specific topics, concepts, or tools in the analyzed literature reflects their representation within the scientific discourse and should not be regarded as evidence of their relative effectiveness or clinical superiority.

Despite these limitations, the sample size and interdisciplinary coverage ensure the representativeness of the analysis. In addition, the identified limitations point to directions for further research, including expanding data sources, considering publications in other languages, and integrating bibliometric methods with clinical and qualitative studies for a more comprehensive understanding of communication and preventable harm. It should also be acknowledged that the proposed five-level communication governance framework was derived from bibliometric findings and remains conceptual in nature. Accordingly, future research should focus on its empirical validation in diverse clinical and organizational contexts, including the evaluation of the reproducibility of the identified levels, their interrelationships, and their practical usefulness for enhancing patient safety strategies.

## 6 Conclusion

Over six decades, research on communication and medical errors has evolved from descriptive accounts of adverse events toward systemic interpretations reflected in the scientific literature.

The bibliometric mapping indicates that communication occupies a structurally central position within the patient safety knowledge network, spanning organizational, team, pharmacotherapeutic, patient-centered, and digital domains. This structural centrality reflects recurrent thematic associations.

Based on large-scale bibliometric patterns and interpretative synthesis, this study proposes a conceptual communication governance framework that organizes these domains into a coherent analytical structure. The framework should be understood as a heuristic model derived from literature patterns. These findings are supported by cross-database validation, which established consistent patterns across Web of Science and Scopus.

In increasingly digital and hybrid healthcare systems, communication reliability may represent an important systemic dimension of patient safety, warranting further empirical investigation.

Future research should develop measurable communication governance indicators, replicate findings across multiple bibliographic databases, and conduct empirical studies evaluating the framework within healthcare management contexts, including nursing practice.

## Data Availability

The original contributions presented in the study are included in the article/Supplementary material, further inquiries can be directed to the corresponding authors.
