# Supplementary material for: Communication and medical errors: a bibliometric analysis of research trends and knowledge structures
Source: Front Med (Lausanne). 2026 Aug 28;13:1856744. doi: 10.3389/fmed.2026.1856744 (PMC13561824; doi:10.3389/fmed.2026.1856744)
Supplement: Supplementary file 1 [file Supplementary_file_1.docx]

Supplementary Material

**Identification of studies via database**

Records removed before screening:

Duplicate records (n = 0)

Automation tool exclusions (n = 4) correction

Retracted publications (n = 7)

Records identified from:

Web of Science Core Collection (n = 13,544)

**Identification**

Records excluded

(n = 102):

Not meeting predefined bibliometric criteria

Records screened

(n = 13,533)

**Screening**

Publications included in the bibliometric analysis

(n =13,431)

**Included**

Supplementary Figure S1. Publication selection scheme (PRISMA) for bibliometric analysis

Supplementary Figure S2. Distribution of publications by subject category in Web of Science (1964–2026)

Supplementary Table S1: Top 10 countries/regions by number of publications in the field of communication and medical errors (1964-2026)

| Country | Number of publications | % of total |
| --- | --- | --- |
| USA | 2 340 | 17.4 |
| England | 701 | 5.2 |
| Australia | 563 | 4.2 |
| Canada | 426 | 3.2 |
| Germany | 356 | 2.7 |
| China | 339 | 2.5 |
| Spain | 220 | 1.6 |
| Italy | 211 | 1.5 |
| Switzerland | 171 | 1.3 |
| India | 169 | 1.3 |

Supplementary Table S2. Top 5 publishers, journals, and organizations by number of publications in the field of communication and medical errors (1964-2026)

| **Entity** | **Record** | **Count (% of 13431)** |
| --- | --- | --- |
| ***Publishers*** | | |
| Elsevier | 1 051 | 7.8 |
| Springer Nature | 703 | 5.2 |
| Wiley | 668 | 5.0 |
| Lippincott Williams & Wilkins | 567 | 4.2 |
| BMJ Publishing Group | 292 | 2.2 |
| ***Journals*** | | |
| Journal of Patient Safety | 252 | 1.9 |
| BMC Health Services Research | 212 | 1.6 |
| BMJ Quality & Safety | 206 | 1.5 |
| BMJ Open | 198 | 1.5 |
| Journal of Clinical Nursing | 133 | 1.0 |
| ***Affiliations*** | | |
| Harvard University | 734 | 5.5 |
| Harvard University Medical Affiliates | 656 | 4.9 |
| University of California System | 437 | 3.3 |
| Harvard Medical School | 424 | 3.2 |
| University of Toronto | 356 | 2.7 |

Supplementary Figure S3. Keyword trends with peak bursts (≥5), bibliometrix, 1964–1999

Supplementary Figure S4. Keyword trends with peak bursts (≥5), bibliometrix, 2000–2010

Supplementary Figure S5. Keyword trends with peak bursts (≥5), bibliometrix, 2011-2019

Supplementary Figure S6. Geographical research networks of countries/regions based on publications in the field of communication and medical errors (Web of Science) 1964–2026
